# Personality, identity, and Artificial Intelligence: a grand challenge

**DOI:** 10.3389/fpsyg.2026.1817687

**Published:** 2026-04-24

**Authors:** Gerald Matthews, Ana-Maria Bliuc

**Affiliations:** 1 Department of Psychology, George Mason University, Fairfax, VA, United States; 2 Division of Psychology, University of Dundee, Dundee, United Kingdom

**Keywords:** artificial intelligence, personality, social identity, sociotechnical system, wellbeing

Modern Artificial Intelligence (AI) is transforming personality research. Personality is expressed in a world in which individual aspirations and social interactions are increasingly mediated by AI, either overtly or covertly (Matthews et al., 2024). There are likely to be dynamic, reciprocal relationships between personality and technology usage. Personality factors such as trust and technophobia influence engagement with AI in work and leisure settings. Conversely, interactions with AI may feed back into stable attitudes, emotional dispositions, and social identities related to AI. Wider societal concerns about the impact of digital technologies such as smartphones on personality and social functioning are heightened by AI. In this Grand Challenge article, we briefly review some emerging research areas and topics that will advance our understanding of how personality and identity interact with AI in the context of technology-driven cultural change.

Previous research has addressed both granular and high-level, “big picture” issues (Matthews et al., 2021). Much extant personality research is granular: it explores how traits influence subjective experience and behavior in well-defined interactions with the physical or social environment. Studies of individual differences in interactions with specific digital systems, such as decision aids and chatbots, can promote constructive human-AI interactions by personalizing the technology to support individuals. The social context is also critical—for example, AI systems are increasingly participating and interacting with social media as autonomous agents. Joint human-AI online communities can have both beneficial and harmful effects, as they can provide large-scale, effective emotional support and widen access to information (O'Leary, 2023). However, they can also create risks as bots can amplify the effects of low-credibility content, exploit confirmation bias, and trigger “information disorders” and extremist dynamics through network effects (Betts et al., 2026; Tomassi et al., 2024).

At a higher level, human-AI interactions take place within rapid cultural changes driven by technology that influence social norms of behavior. The nature of work is changing as physical and cognitive activities are offloaded to AI, and individuals are hired, fired, and monitored by AI (e.g., Bankins et al., 2024). Similarly, social relationships are increasingly structured around online communication, smartphone apps, and AI technology. Research on human–machine systems shows that even peripheral AI agents can shape collective outcomes. Importantly, these outcomes arise from interdependent human–human and human–machine *interactions*, not from isolated actors (Tsvetkova et al., 2024). This means that a new challenge for social psychology is to treat AI as a *social agent* and theorize agency, trust, and moral influence in multi-agent (hybrid) systems. Because these hybrid systems are embedded within broader cultural ecologies, identity processes are simultaneously shaped by evolving social norms and collective narratives. Online identities are shaped by dynamic cultural trends, and how such trends play out will likely differ according to pre-existing cultural differences within and between nations.

Research areas also differ in their focus on the individual vs. the social construction of identity. At the individual level, research builds on existing personality trait models, human-computer interaction (HCI) research, and applied cognitive psychology to investigate how individuals vary in their usage of AI-powered technologies and in their reactions to them. Beyond individual differences in human–AI interaction, personality and social psychology must address *identity*. At the micro level, AI is now embedded in everyday settings and shapes how identities are activated and regulated through feedback. At the macro level, AI is part of the sociotechnical infrastructure through which collective meaning, moral values, and group boundaries are constructed, negotiated, and refined.

Although research on personality, identity, and AI is thriving, it is often scattered and disconnected. Here, we aim to promote the coherence of research by identifying major research areas differentiated by the twin axes of (1) granularity vs. high-level, “big picture” issues and (2) individual differences vs. social identity. Within this 2 × 2 scheme, we briefly define key research questions and new approaches to understanding personality in the digital age.

## Individual differences in interactions with AI

Established personality dimensions such as internet anxiety, technophobia/philia, and computer self-efficacy shape attitudes, emotions, and behaviors toward conventional computer systems (Matthews et al., 2021). However, the fundamental differences between conventional digital systems and modern AIs will change the role of personality in digital interactions. These differences include the vast cognitive capabilities of AI, social agency and natural language communication, the assumption of a consistent human-like persona, and a relationship history with the user. New scales for constructs such as trust in AI, humanlikeness, and social presence are proliferating (Esterwood et al., 2021). However, scale development is in its infancy. The quality and validity of these scales vary greatly, and there is no overarching psychometric model to support the integration of research findings.

Research on the sources, development, and malleability of individual differences is also lacking. Concerns about the effects of smartphone use and social media on child development (Haidt, 2024) often neglect the nuances of individual differences in vulnerability to harmful impacts (Matthews et al., in press). Longitudinal studies of the development of attitudes toward AI are needed in both children and adults, because current research on new personality constructs is typically cross-sectional. Understanding the role of established, biologically-based traits such as the Big Five is essential, but some novel constructs, such as online personas, are less stable and more malleable than conventional traits (Olivero et al., 2020).

A third research focus is the consequences of attitudes and emotions toward AI. Which personality factors influence offloading significant life decisions to AI, treating an AI as a friend or confidant, or allowing an AI agent to manage one's social media interactions? These questions can be addressed through experimental studies and prospective studies examining how individual differences interact dynamically with AI usage.

## Stress and wellbeing in the age of AI

The sociotechnical perspective on the impact of AI positions the individual within interacting technological, organizational, and cultural systems (Matthews et al., 2025; Yu et al., 2023), suggesting multiple goals for personality research. First, AI introduces novel threats such as uncertainty over the basis for AI judgments, social stressors associated with human-like interactions with AIs, and loss of self-esteem when AIs surpass humans in cognitive capabilities and decision-making authority (Matthews et al., 2024). There is an overarching threat to the person's connection to reality or epistemic rationality. Maintaining well-being and a sense of cohesion depend not only on basic and AI-linked traits but also on socioeconomic status and the psychosocial environment (Brunner and Marmot, 2006). How will changes in social organization driven by AI interact with traits to produce stress at the individual and group levels?

Second, AI enables both benefits and harms (e.g., Bond et al., 2025). AI can potentially improve quality of life by freeing individuals from mental drudgery, providing decision support based on a vast knowledge base, and powering therapeutic interventions. However, its knowledge base is contaminated by human biases, including racial and gender biases. Its outputs are frequently unexplained and unverifiable. It is indifferent to privacy, and it is readily utilized for malicious purposes, such as cybercrime and disseminating misinformation. In organizations, AI enables practices such as intrusive surveillance and algorithmic hiring and firing that dehumanize work relationships. Personalized alignment of AI to match the user's personality, skills, and values can enhance its usefulness and widen access but also risks infringing on privacy and reinforcing bias (Kirk et al., 2024). Personalized alignment is more than a technical design issue. It raises difficult questions about how personalization should be bounded and who should decide on the principles that define those bounds (Kirk et al., 2024).

Third, the usage and impact of AI reflect fluid and sometimes contested cultural norms, such as the level of government regulation of AI that should be implemented. There is also a growing body of literature on cross-cultural differences in attitudes toward AI and how these vary across cultural dimensions, such as individualism vs. collectivism (Barnes et al., 2024). Another research challenge is that AI's increasing presence in our lives is likely to lead to new human cultures unlike anything in history, requiring emic perspectives on the attributes of personalities tied to digital culture to complement universal, etic models (Matthews et al., 2021).

## How does AI reshape the formation and regulation of identity in everyday social environments?

AI systems do not merely deliver information but increasingly function as interactive social actors, as they respond, adapt, and mirror users, creating feedback loops that contribute to the activation, validation, and regulation of social identities (Metzler and García, 2023; Pedreschi et al., 2025). Through these interactive processes, AI-mediated environments curate, amplify, and stabilize collective narratives that provide meaning, relevance, and moral orientation to group members (Bliuc et al., 2024; Sartori and Theodorou, 2022). These developments pose a theoretical challenge to our field because classic theories in social psychology assume that identities are shaped by interaction with other individuals, groups, and social institutions (Spears, 2021). However, AI introduces a novel “interaction partner,” one that is highly responsive, personalized, scalable, and opaque, yet embedded in everyday communication, community participation, and information access. Existing theoretical models are not well-equipped to explain how identity dynamics operate when group-relevant narratives are curated or generated by adaptive systems. A central research question is whether AI-driven adaptation stabilizes social identities (perhaps by providing coherence, validation, and belonging) or narrows them by reinforcing exclusionary narratives and reducing identity flexibility. A related question concerns wellbeing—for example, under what conditions do AI-mediated collective narratives support meaning, relevance, and psychological resilience, and when do they contribute to dependency or entrenchment? AI-augmented online communities provide an important test case. In contexts such as addiction recovery or mental health support groups, AI systems may facilitate positive identity change by supporting new recovery narratives (Li et al., 2023; Thakkar et al., 2024), but they may also reinforce static or dependent identities, posing additional risks(Elyoseph et al., 2024). Importantly, issues such as trust, vulnerability to misinformation, and polarization should be analyzed not only as cognitive biases but also as identity-regulatory processes operating through perceived group alignment and collective meaning-making (Efstratiou and De Cristofaro, 2022).

## AI as identity infrastructure: meaning, moral values, and boundaries

AI systems in conjunction with online media are becoming a part of the infrastructure through which collective identities are constructed, represented, and evaluated. They can mediate how individuals and groups are seen by others, how collective narratives circulate and evolve, and how meaning and moral values are articulated and contested (Gerbaudo, 2022). Psychology currently lacks models of identity and agency for conditions in which aspects of identity are delegated to non-human agents (e.g., use of avatars, digital twins, and so on), personal and interaction data persist as identity traces, and social recognition is mediated by algorithmic systems rather than interpersonal or institutional processes (Bonnefon et al., 2023). These developments raise important questions about how collective meaning and moral order are sustained, both now and in the future. One risk concerns identity delegation and representation. When AI systems generate profiles, narratives, or predictions about individuals or groups, they relocate control over identity representation to system designers and other (ambiguous) operators, raising questions about agency, power, and voice. A second risk concerns collective meaning and moral values. Attitudes toward AI, privacy, and technological change often reflect group-based values and shared (ideologically bound) narratives rather than being driven by deliberative ethical reasoning, with implications for intergroup relations and polarization. A third risk concerns power and inequality, as AI-mediated systems may differentially amplify, normalize, constrain, or erase particular identities. If personality and social psychology fail to theorize identity, meaning, and moral boundaries under AI mediation, then we will lack empirically grounded and theoretically informed frameworks to detect, evaluate, and mitigate the potential engineering of identity by platforms, corporations, and state actors.

## Conclusion

The AI revolution has stimulated a creative ferment of new research in personality and social psychology. We advocate for a more systematic approach than currently exists that centers on well-defined research questions related to individual differences in interactions with AI and the negotiation of identity in communities mediated by AI. Table 1 summarizes some major topics for research that we have briefly discussed. These are not intended to be exhaustive, but they illustrate some areas open to focused investigation. There is also a need for multidisciplinary approaches that include perspectives from computer science, neuroscience, communication science, and sociology. We encourage researchers interested in these issues to consider the Personality and Social Psychology section of *Frontiers in Psychology* as an outlet for their studies.

**Table 1:** A selection of research goals differentiated by granularity/big picture and individual differences/social identity perspectives.

	Granular	Big-picture
Individual differences in human-AI interaction	•Constructing psychometrically rigorous dimensional structures to integrate new constructs with existing trait models •Determining the sources, development, and malleability of personality factors linked to AI •Determining the consequences of personality factors for usage and engagement with AI	•Investigating vulnerability and resilience to the novel stressors of the digital age from a sociotechnical perspective •Personalizing AI at work and in other contexts to optimize the benefits-to-harms ratio at the individual and societal levels •Investigating how multiple aspects of culture shape a person's interactions with AI
Construction of identity	•Identifying the mechanisms responsible for AI feedback loops (i.e., activating, stabilizing, or narrowing down social identities) •Examining how algorithmic curation shapes perceived group norms and narratives •Investigating identity flexibility vs. entrenchment under AI feedback loops •Examining trust, information disorders, and polarization as processes that regulate identity	•Theorizing identity delegation and representational authority under hybrid interactions •Understanding how AI systems shape moral boundaries and drive polarization •Investigating power asymmetries in AI-mediated identity amplification or erasure •Developing psychological, ethical, and moral frameworks for identity, agency, and representation in sociotechnical systems.
